# A “light chaser” and his dream of Optics Valley of China ——To commemorate Prof. Dexiu Huang

**DOI:** 10.1007/s12200-022-00053-0

**Published:** 2022-12-28

**Authors:** Wei Hong, Zhen Wang, Jianji Dong

**Affiliations:** Wuhan National Laboratory for Optoelectronics, Huazhong University of Science and Technology, Wuhan, 430074 China

**Fig. 1:**
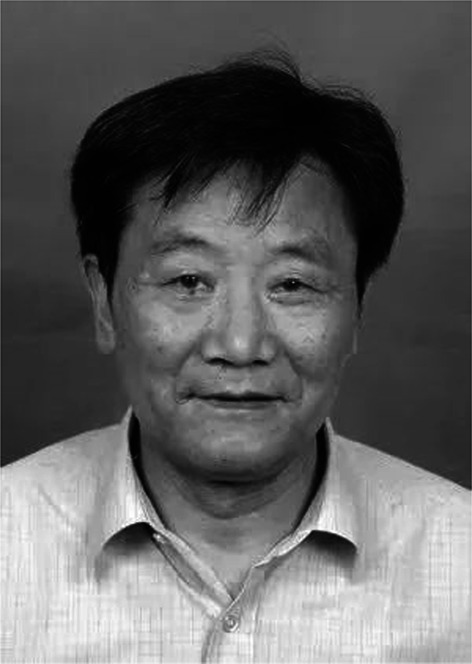
Dexiu Huang

Dexiu Huang (黄德修, 1937–2022) (Fig. 1) was a professor and doctoral supervisor in the School of Optical and Electronic Information, Huazhong University of Science and Technology (HUST), China. He was also the former director of the Department of Optoelectronic Engineering (now the School of Optical and Electronic Information), the Dean of the School of Information, and the deputy director of Wuhan National Laboratory for Optoelectronics (WNLO) (preparatory). Prof. Huang was a pioneer in the field of optical communication in China. He made outstanding contributions in the field of optoelectronic devices, and was the first proponent of the “Wuhan Optics Valley of China (OVC)”. He was given a “Young and Middle-aged Experts with Outstanding Contribution in Hubei Province” award, and was the “National Model Teacher” and “Model Worker of the National Education System” awarded by the Ministry of Education and the former Ministry of Personnel. He was awarded the “National May Day Labor Medal”, and was selected as one of the “30 Innovative People in 30 Years of OVC”.

On October 14, 1937, Dexiu Huang was born in Ningxiang County, Hunan Province, China. His early education path was extremely tortuous. He had dropped out of school several times due to poverty and other reasons. When he graduated from junior high school in 1954, he was already 17 years old. After graduation, he went to Shenyang Electric Power Technical School of the Northeast Electric Power Administration. In 1955, he became a worker in Huangshi Thermal Power Plant. He studied hard and worked hard, and was soon promoted to become a fourth level technician. However, the disillusionment of not realizing his dreams of going to college was still a deep regret in his mind. Encouraged by his colleagues, he finally decided to change his destiny after a fierce struggle. He studied by himself in his spare time, and finished the high school courses in only one and a half years. In 1958, he attended the national college entrance examination, and was admitted to the Department of Electrical Engineering of Huazhong Institute of Technology (now HUST). majored in Electrical Machinery, thus beginning a new chapter of his life. Although he had not attended high school and went to college only through self-study, he always ranked among the top students due to his hard work and strong self-study ability. In 1960, Huang transferred from the Department of Electrical Engineering to the Department of Radio Electronics to study radio electronic materials and components. In 1963, he graduated with distinction from Huazhong Institute of Technology, and became a faculty upon graduation.

In 1972, Huang was transferred from Department II of Radio Electronics (i.e., the Department of Electronics) to the newly established laser research team and engaged in research on solid-state lasers. After years of efforts, he and his colleagues successively developed ruby pulsed lasers, neodymium glass pulsed lasers, Nd:YAG continuous-wave lasers, and conquered a number of technology challenges such as Q-switching and mode-locking. They also put laser welding and laser medical treatment into practice. After 1978, with the improvements made in the area of solid-state lasers, and high-power carbon dioxide lasers, the research achievements and discipline construction of Huazhong Institute of Technology in the field of energy optoelectronics had gradually become prominent in China. However, Huang and his team were well aware that the research of energy optoelectronics in China was still far behind that in the United States.

In September 1981, Huang went to Oregon Graduate Center (OGC) as a government-sponsored visiting scholar for two years. After arriving at OGC, he was first assigned to study laser speckles. But after visiting some laboratories and gaining deeper understanding, he developed a strong interest in semiconductor photoconductive switches, which was on the research plan of Prof. Richard A. Elliott. He felt that this technology would be developed rapidly and deeply affect the world in the near future. After careful consideration, Huang resolutely chose to study semiconductor photoconductivity and was finally permitted to do so by OGC and his supervisor. After more than a year of unremitting efforts, Huang achieved good results in semiconductor photoconductive switches. He made an oral report at the 1982 annual meeting of the Optical Society of America (OSA) [1], and further research results were published in Applied Physics Letters in 1993 [2]. In the spare time during his research, Huang often went to the OGC Library, digging into a large quantity of literature relating to semiconductor lasers, semiconductor photodetectors and optical properties of semiconductor materials. In his view, the field tests of optical telecommunication in the United States in 1975, and short distance optical telecommunication tests in the late 1970s, fully evidenced the great potential of optoelectronics in information field. He expected that optical fiber communication would have bright prospects because of its huge bandwidth, confidentiality and reliability, and semiconductor optoelectronic devices were the key to the development of optical fiber communication.

After returning to China in February 1983, Huang was assigned to continue leading laser research. At that time, he was determined to take a step forward in the new field of information optoelectronics represented by optical fiber communication. Starting with semiconductor optoelectronic devices, he opened up the road to “chase after light”. In 1983, research on optical fiber communication in China had just started. Wuhan Research Institute of Posts and Telecommunications (WRI, now part of the China Information and Communication Technology Group Co., Ltd.) had just completed the first 13 km optical fiber communication test line across the three towns of Wuhan. After difficult lobbying, Huang obtained support from WRI and the 6th Five Years Programs for Science and Technology Development of China (6th Five Years Program), and began to study semiconductor optical amplifiers (SOAs), which was thought to be a candidate for relay amplifiers in long-distance optical fiber communication systems.

At that time, optical fiber communication was still a new topic. The first problem Huang encountered was that it was very difficult to find like-minded researchers to form a research team. At the beginning of 1984, Huang successfully persuaded Deming Liu (刘德明), who was then a postgraduate student just graduated from Chengdu Institute of Telecommunication (now the University of Electronic Science and Technology of China), to join his team. Later, two postgraduate students who majored in laser technology, Mi Zhou (周宓) and Xuefeng Liu (刘雪峰), also asked to join his team. A vigorous and effective research team was then formed. As the team leader, Huang devoted endless effort to his team and his research. He took on many tasks, ranging from sorting and copying an extensive range of literature to designing research routes. As a pioneer in the field of optoelectronics he had to “feel the stones”, and led his team to carry out research on SOA day and night. As their research advanced, they obtained important results not only in Fabry-Pérot SOAs (FP-SOAs) that was studied in the early stage of their research, but also in traveling-wave SOAs (TW-SOAs) that had more practical value.

Huang and his team successfully completed the task of the 6th Five Years Program and received commendation from the State Scientific and Technological Commission of China (now the Chinese Ministry of Science and Technology). They also engaged with the 7th Five Years Program to continue their research. In 1987, the National High Technology Research and Development Program (863 Program) was officially launched. After a sudden investigation by experts from the Committee of Experts in the Field of Information for the 863 Program, Huang’s research topic was funded by the 863 Program. With the dual support from the 7th Five Years Program and the 863 Program, the innovation potential of Huang’s team was eventually unleashed.

**Fig. 2:**
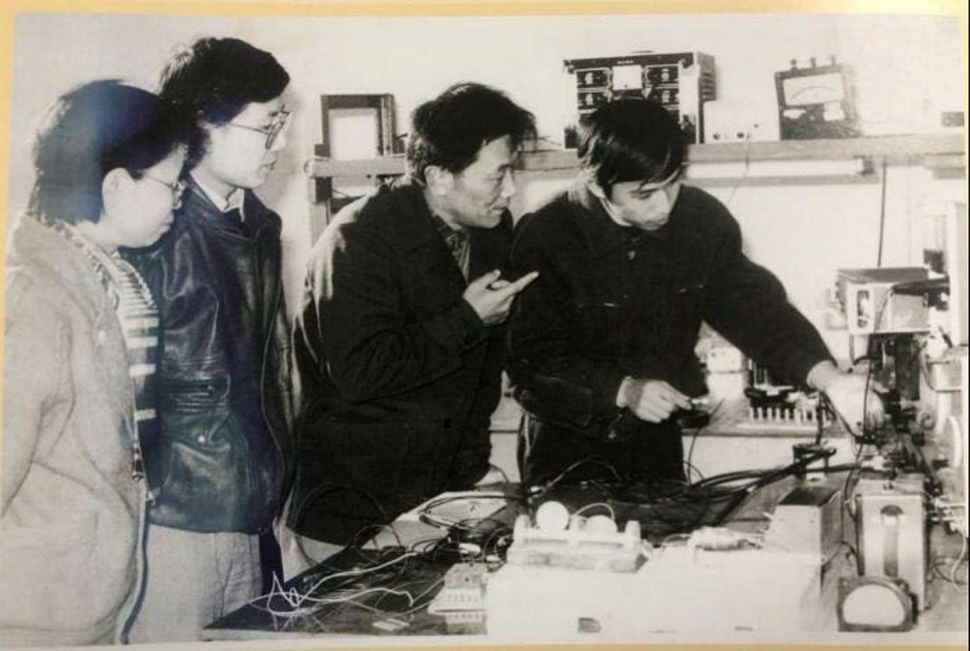
Dexiu Huang (second from right) conducting research in the laboratory

In December 1991, the new TW-SOA with single-ended fiber coupling developed by Huang’s team won the third prize of the National Invention Award, and Huang’s team was honored as an “innovation team” by the State Scientific and Technological Commission of China. Figure 2 shows Huang working in the laboratory. From 1992 to 1996, Huang entered the Expert Group in Optoelectronics Field for the 863 program, which enabled him to have a broader view of the cutting-edge topics of optics and optoelectronics.

With the thorough research of SOA and the breakthrough of erbium-doped fiber amplifiers in the late 1980s, researchers realized that SOA may not be suitable for wavelength division multiplexed optical transmission due to gain saturation effect and large noise figure. The enthusiasm for SOA began to ebb away. Huang, however, believed that it would be possible to realize multiple signal processing functionalities in the optical domain by exploiting gain saturation and other nonlinear effects of SOA. Under his guidance, Xinliang Zhang (张新亮), his doctoral student, began to explore wavelength conversion based on SOA. Later, the research on wavelength conversion was funded by the 863 Program. When the Chinese National Basic Research Program (973 Program) was launched, Huang joined the research team led by Prof. Yi Luo (罗毅) of Tsinghua University. He undertook a sub-project of the 973 Program, and continued the research on wavelength conversion. In the next 20 years or so, his team studied improvement of SOA performance for the requirements of optical signal processing [3–12], and carried out research on SOA-based wavelength conversion [13–22], signal regeneration [23–27], logic gates [28–42], format conversion [43–47], and microwave photonics [48–56]. In 2003, Huang and his team won the first prize of the Science and Technology Award of the Ministry of Education for their outstanding contributions to the theoretical research and application of SOA based on energy band tailoring.

In 2005, Huang was nearly 70 years old, but he was still exploring the frontiers of science and technology. In his view, electrons and photons are a pair of microscopic particles with obvious parallelism and complementarity in nature. Similar to electronics, integration was also inevitable direction of photonics. Drawing on the successful practice of CMOS integrated circuits and some exploration of photonic integration over the years, he summarized the technical key points of optoelectronic integration: (1) Large scale integration of optoelectronic devices should rely on planar processes with lithography as the core technology, because only planar processes can make it possible to fabricate different functional devices or arrays of the same devices on the same substrate. (2) Semiconductor materials are the most suitable for optoelectronic integrated circuits. Due to the functional diversity of photonic devices, the road to optoelectronic integration was a complicated process of material selection and optimal combination. He believed that demand-pull and technology-push provided the law for the continuous development of science and technology. In view of the strong demand for optoelectronic integration brought by optical interconnection, optical switching, etc., there was a need for some related technologies. Therefore, it was necessary to explore some technologies that were based on semiconductor planar processes and could form device functions during material processing, so as to realize systems on-chip with different integration scales. Under his leadership, his team began to explore new fields such as micro-ring resonators [57–67].

After initiating the research on optical fiber communication and semiconductor optoelectronics in HUST, Huang attached great importance to discipline development and talent cultivation. For him, training high-quality talents was not only needed for the development of his team, but also an important cornerstone for serving the strategic development of our country. At the end of 1987, under the impetus of Huang, the Department of Optoelectronic Engineering, HUST set up the specialty of Optical Fiber Communication. The Optical Fiber Communication teaching and research section was established at the same time, and Dexiu Huang and Deming Liu served as the chief and deputy directors respectively. From the students of Grade 85 in the department, 15 students were selected to be the first majoring in optical fiber communication after voluntary application. Huang and his team set up several specialized undergraduate courses, including “Semiconductor Optoelectronics” lectured by himself, “Fiber Optics” lectured by Deming Liu, and “Optical Fiber Communication System” lectured by Siyuan Yu (余思远). Experimental courses and internships were also arranged as indispensable teaching steps.

In 1985, Huang proposed the topic of “Semiconductor Optoelectronics” as the textbook for undergraduate and graduate students. His submitted manuscript was eventually selected by the textbook editing committee of the former Chinese Ministry of Electronic Industry and was published by the University of Electronic Science and Technology Press in 1989 [68]. In 1992, this textbook was awarded the first prize of “Excellent Textbook” by the Ministry of Electronic Industry. Later, it was republished twice, in 2013 and 2018 [69, 70]. The textbook Fiber Optics, written by Deming Liu, was also published as a national planning textbook.

**Fig. 3:**
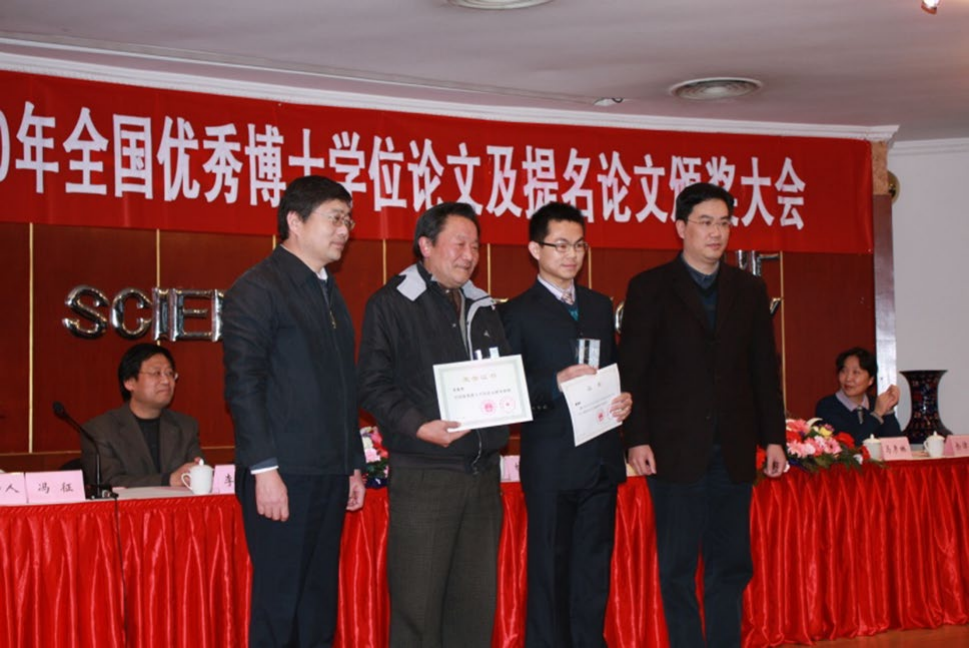
Jianji Dong (董建绩, second from right), a doctoral student of Huang (second from left), won the National Excellent Doctoral Dissertation Award in 2010

Huang’s team made rapid progress during the complementary development of research and teaching, and made positive contributions to the rise and growth of the optoelectronic information discipline of HUST. In his 60 years teaching career, Huang published 9 monographs and textbooks and supervised 142 doctoral and master’s students. His students won the “National Excellent Doctoral Dissertation Award” and other honors several times (Fig. 3). In 1998, Huang was selected as one of the ten winners of the first “Bole Prize” of HUST. He was awarded the “National Model Teacher” and “Model Worker in the National Education System” by the Ministry of Education and the former Ministry of Personnel in the same year. In 2001, He won the National May Day Labor Medal. But Huang said, “In fact, the provincial and national honors I won were actually based on the work of my team.”

**Fig. 4:**
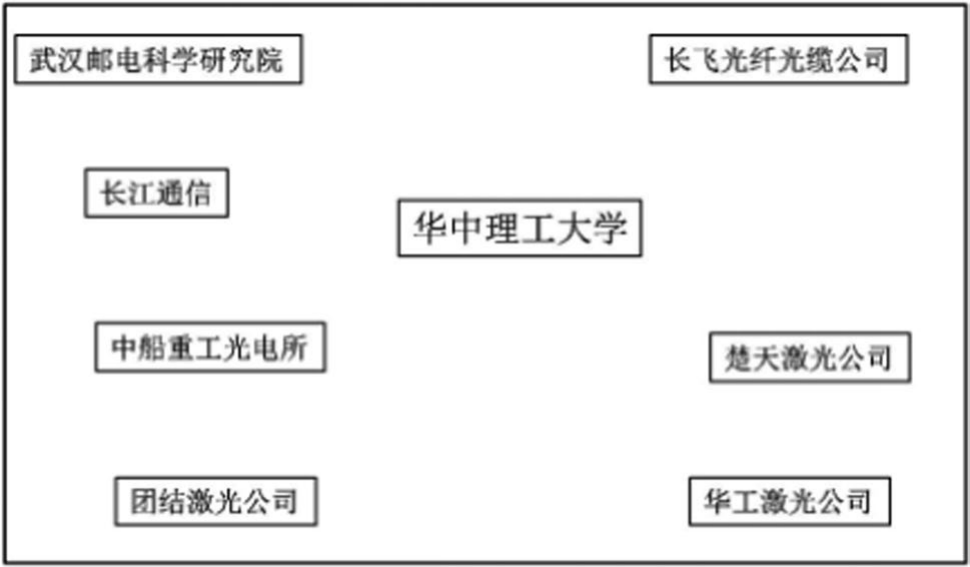
Overview of some influential optoelectronic-related enterprises in Wuhan shown by Huang during the nine-day visit to Taiwan, China in 1998

**Fig. 5:**
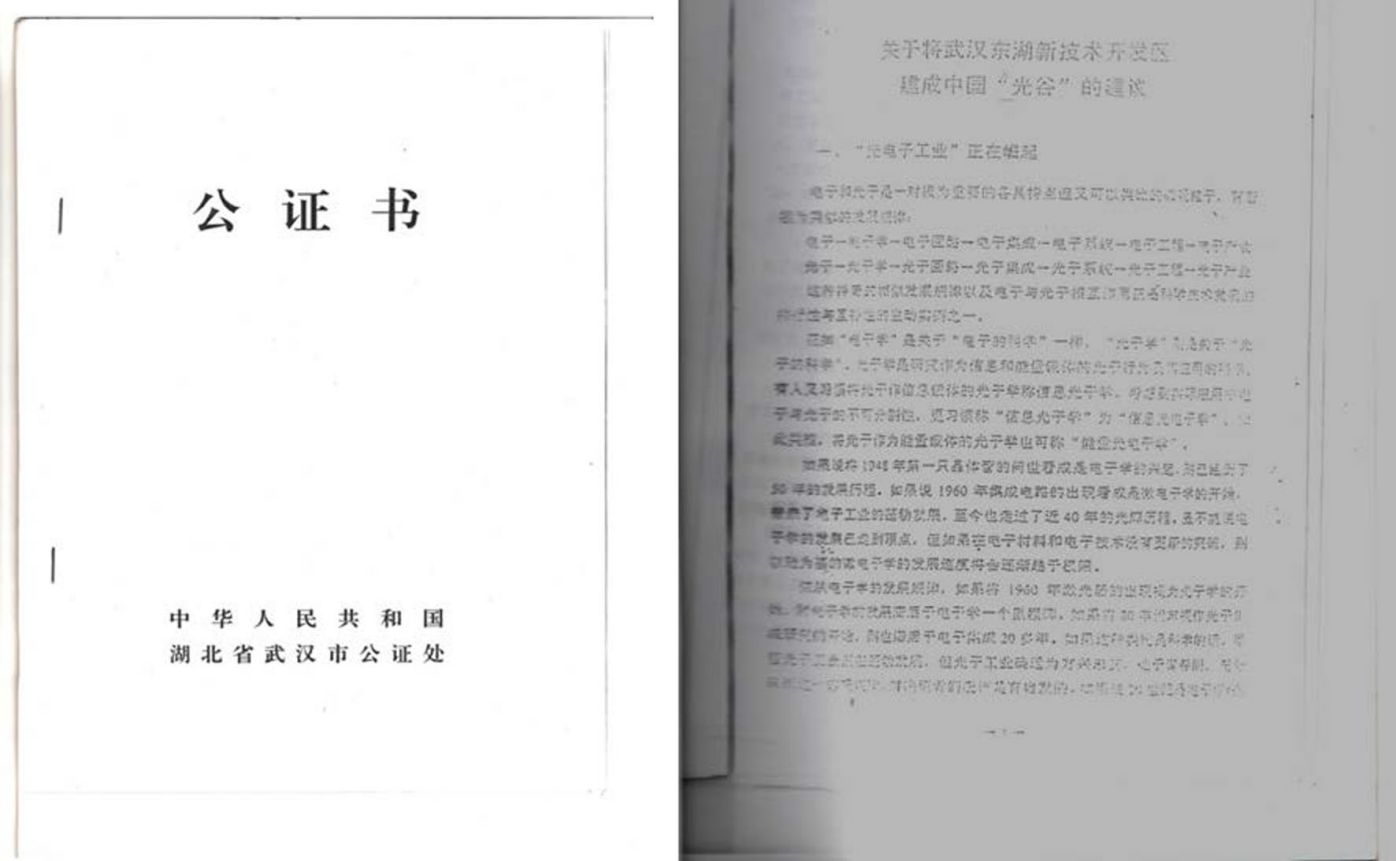
Proposal on constructing OVC in the Wuhan East Lake High-Tech Development Zone submitted by Huang in 1998

In the late 1990s, the optoelectronic information industry, represented by optical fiber communication, was booming. In July 1998, Huang participated in a delegation organized by the Natural Science Foundation of China and made a nine-day visit to Taiwan, province of China at the invitation of Taiwan Photonics Industry & Technology Development Association (PIDA). During the visit, he went to the Industrial Technology Research Institute in Hsinchu, the Hsinchu Science Park, Taiwan Tsing Hua University and Taiwan Chiao Tung University, Taiwan University and Cheng Kung University. He also went to some research institutes engaged in optical fiber communication and a number of enterprises producing LED devices or optoelectronic materials, such as LITEON Group and OPTOTECH. He was deeply impressed by the booming research and development of optoelectronics in Taiwan, China. During the exchange between scholars on the last day of the visit, Huang shared a schematic picture of the distribution of some influential optoelectronic-related enterprises in Wuhan (Fig. 4). These enterprises were located around HUST, and some of them, such as HGTECH, CHUTIAN Laser and UNITY Laser, were actually founded by graduates from HUST. In addition, more graduates from HUST had become senior managers or the technical backbone of these surrounding optoelectronic enterprises. While showing this picture, Huang suddenly realized that it was very similar to Silicon Valley in the United States. This became the inspiration for his proposal of Optics Valley of China (OVC). With the support of Ji Zhou (周济), the president of HUST at that time, Huang submitted a proposal to the Wuhan Municipal Government in December 1998 on behalf of HUST for the construction of OVC in the Wuhan East Lake High-Tech Development Zone (Fig. 5). Thus, Huang is considered as the first proponent of OVC.

On the eve of the Chinese New Year in 2000, Huang was invited to attend a reception organized by the CPC Wuhan Municipal Committee and Wuhan Municipal Government. At the reception, Huang said: “In the next 50 years, the development of Wuhan would depend on optoelectronics!” His words were extremely powerful and enlightening. In 2000, 13 CPPCC members from Hubei, including Qizhen Xu (许其贞), called for the construction of OVC in Wuhan at the CPPCC National Conference. At the same time, Hubei Province, Wuhan City and the Wuhan East Lake High-Tech Development Zone determined to give the optoelectronics industry the top priority. In 2001, the proposal of OVC was approved by the former State Planning Commission of China and the Chinese Ministry of Science and Technology, and then the construction of OVC started.

**Fig. 6:**
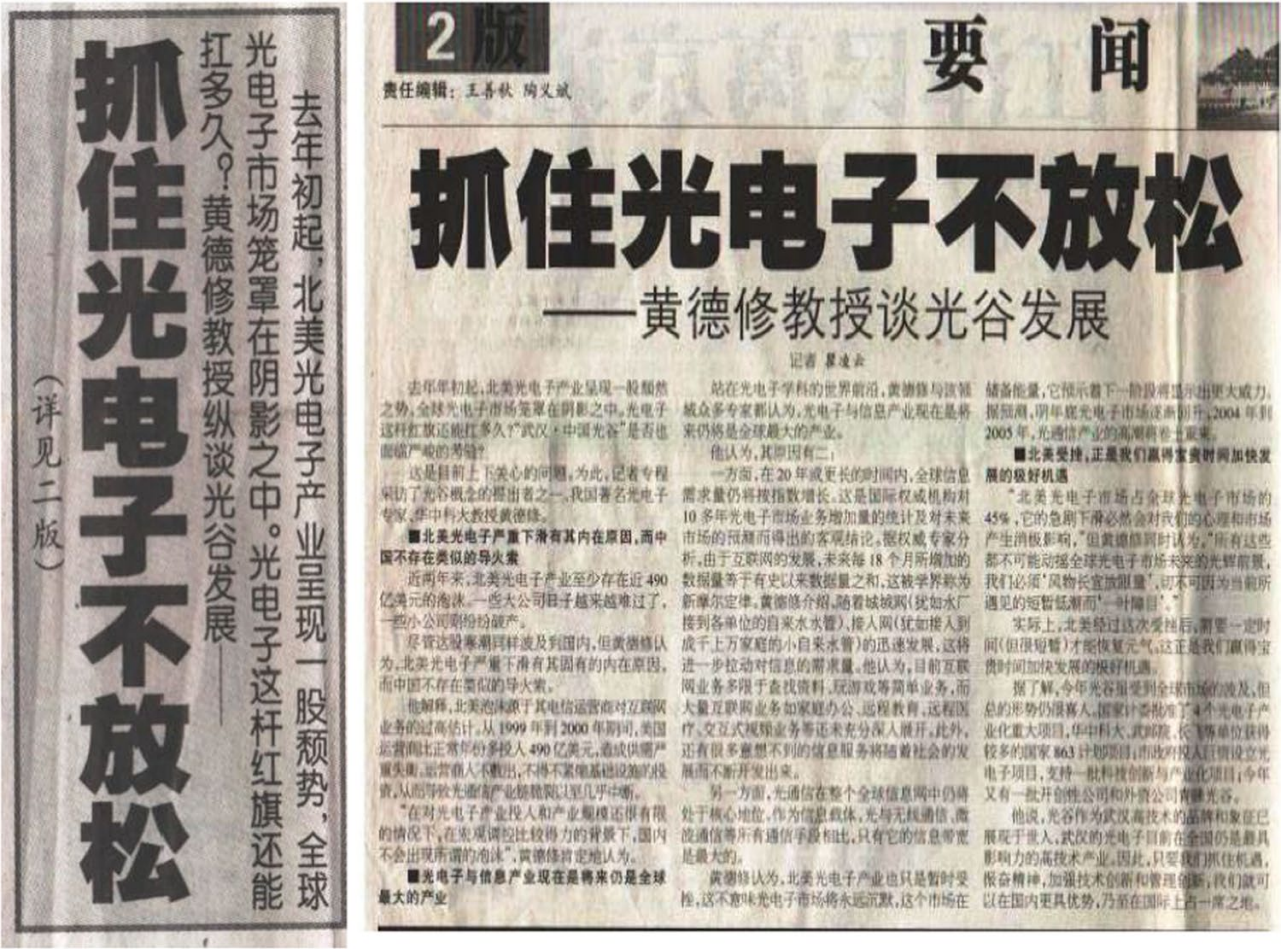
Huang’s report, entitled as “*Grasp optoelectronics and hold on to it”* was published on the first page of *Changjiang Daily* on October 23, 2002

However, due to the imbalance between supply and demand of optical-communication-related products, a large number of optoelectronics companies in some developed countries, such as the United States, had closed down one after another since the end of 1999. This international background was undoubtedly a severe test for the OVC that just started. However, Huang still firmly believed that the international IT bubble could not conceal the objective trend that the optoelectronics industry was bound to greatly develop. He wrote a report, entitled “*Grasp optoelectronics and hold on to it”* to Qingquan Luo (罗清泉), who was Secretary of Wuhan Municipal Party Committee at that time. This report was later published in *Changjiang Daily*, after some modifications (Fig. 6). Under the active advocacy of Huang and the strong promotion of Secretary Qingquan Luo and Ji Zhou, the Vice Minister of the Ministry of Education at that time, the CPC Hubei Provincial Committee and Hubei Provincial Government made an important strategic decision to build OVC, backed by the strength of both Hubei Province and Wuhan City. Today, there has been a fairly solid consensus that optoelectronics is a good theme for the development of the Wuhan East Lake High-Tech Development Zone, as evidenced by the vigorous development of this Zone in the past 30 years.

Regarding the future development of OVC, Huang offered the following outlook in one of his lectures: The development potential of optoelectronics and microelectronics was far from being fully realized. Quantum communication, quantum computing and artificial intelligence were just beginning. Large-scale integration of optoelectronic devices had not been fully realized, although it had been studied for a long time. And there was still large room for improving the cost-effectiveness of some existing optoelectronic devices and systems. The development of optoelectronics still had a long way to go. Photons and electrons and their combination can provide us with huge and long-term potential for innovation. As long as we keep to this point, OVC will always maintain its vitality and continue to create miracles in the world. As the proposer of the concept of OVC, Huang was selected as one of the “30 Innovative People in 30 Years of OVC” in 2018.

**Fig. 7:**
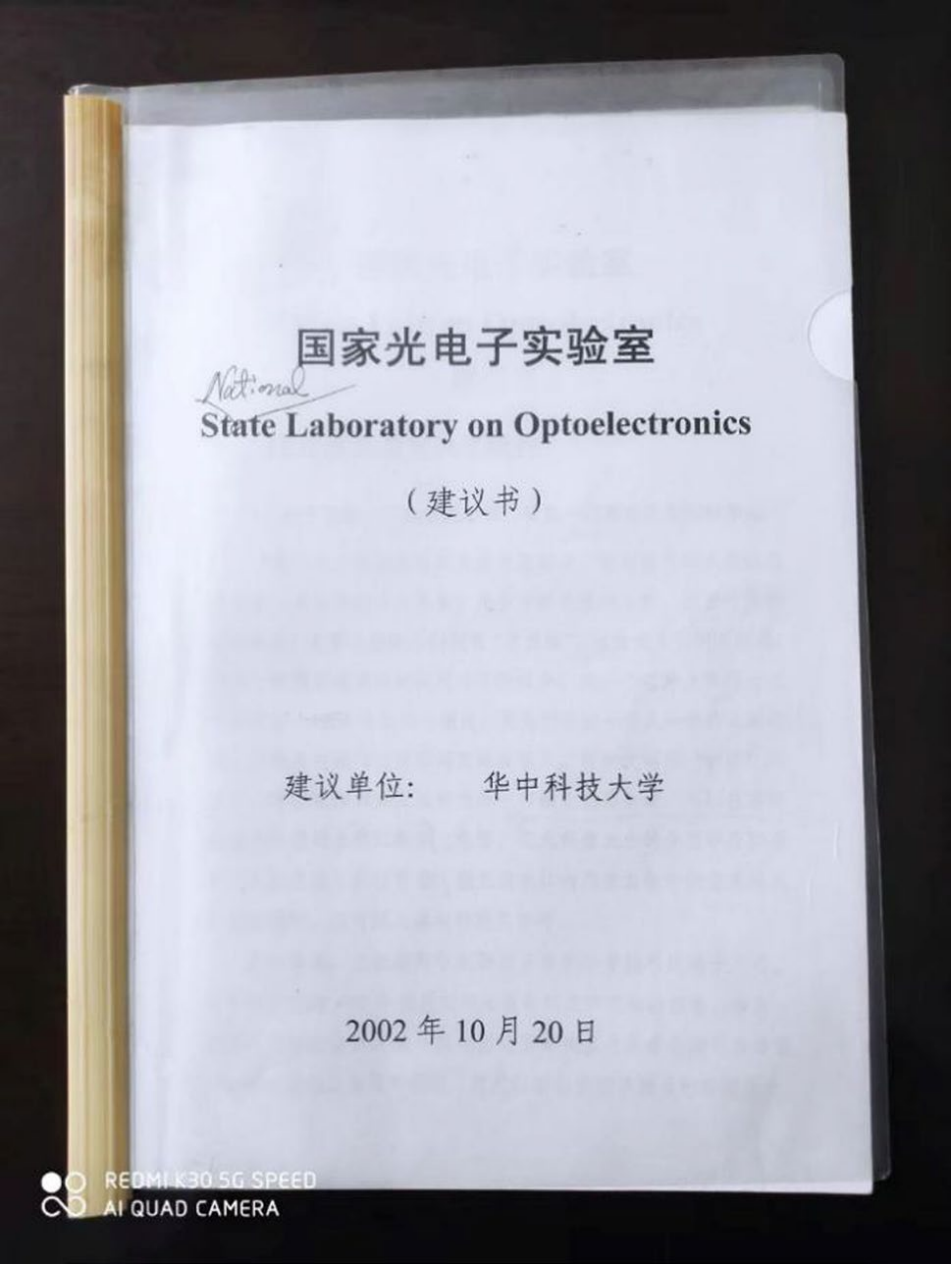
Proposal for constructing Wuhan National Laboratory for Optoelectronics

In order to provide strong scientific and technological support for the sustained development of OVC, the Advisory Committee of OVC proposed to construct a National Research Laboratory for Optoelectronics in Wuhan in 2002 (Fig. 7), and this proposal was submitted to the Ministry of Education and Ministry of Science and Technology. Huang was one of the participants and promoters of the proposal. On November 25, 2003, the Ministry of Science and Technology officially approved the establishment of Wuhan National Laboratory for Optoelectronics (WNLO). In January 2004, the preparatory working group of HUST for WNLO was established, and Huang was the deputy group leader. In April 2004, Huang became the deputy director of WNLO. Researchers engaged in the related areas of optoelectronics from HUST and other partnership institutions were gathered, and eight research divisions were formed: Fundamental Photonics, Laser Science & Technology, Optoelectronic Devices and Integration, Nanophotonics and Microsystems, Data Storage System, Optical Communication Systems and Intelligent Network, Biomedical Photonics, and Space Optoelectronic Technology [71]. Huang personally invited a large number of scientists who had just returned from overseas to join WNLO. The establishment and development of WNLO injected new vitality into the coordinated and sustained development of OVC.

In 2007, the Higher Education Press (HEP) launched the *Frontiers* series of journals. At first, *Frontiers of Optoelectronics* (FOE) was not in the HEP’s plan. However, from the perspective of discipline development in HUST, the president of HUST at that time, Peigen Li (李培根), took the initiative and suggested to HEP that they should launch a journal on optoelectronics. At the invitation of Peigen Li, Huang promoted and completed the preparatory work for the establishment of the journal. Huang fully agreed with Peigen Li’s idea of establishing a journal in the field of optoelectronics. He had mentioned several times that, just as the Bell Labs in the United States had *Bell Labs Technical Journal,* which had high impact in the optoelectronics community, WNLO should also host a prestigious journal, with the aim of becoming an internationally renowned academic center in the field of optoelectronics. Therefore, FOE journal was founded in 2008. Huang invited Bingkun Zhou (周炳坤) from Tsinghua University as the founding Editor-in-Chief, and he himself served as one of the founding Deputy Editors-in-Chief. In order to gain high quality manuscripts, Huang always personally invited experts in the related research area to submit to FOE when he attended academic conferences, or asked the editors of FOE to visit some of his friends with a personal letter hand-written by him. He also reviewed manuscripts himself and guided the editorial work. Even after he stepped down as the Deputy Editor-in-Chief, he still made constructive suggestions for the development of FOE.

In 2008, Huang completely retired from his job. After his retirement, Huang still paid attention to the development trends in the field of optoelectronics and was concerned about the development of the optoelectronics industry in Wuhan. He was often invited to give lectures on different occasions, talking about his experiences of study and scientific research, the development of the optoelectronic information discipline, the past, present, future of OVC, and his dream of OVC. On November 8, 2022, Huang passed away.
